# Genome-wide identification and characterization of SPX-domain-containing protein gene family in *Solanum lycopersicum*

**DOI:** 10.7717/peerj.12689

**Published:** 2021-12-22

**Authors:** Chunwei Li, Qiuye You, Panfeng Zhao

**Affiliations:** 1 Nanchang Normal University, Nanchang, China; 2 Shanghai Center for Plant Stress Biology, Shanghai, China

**Keywords:** SPX, Gene family, Genome-wide analysis, Phosphate ions (Pi) nutrition, *Solanum lycopersicum*

## Abstract

The SYG1, PHO81, and XPR1 (SPX) domain is named after the suppressor of yeast gpa1 (Syg1), yeast phosphatase (Pho81) and the human Xenotropic and Polytrophic Retrovirus receptor1 (XPR1). SPX-domain-containing proteins play pivotal roles in maintaining phosphate ions (Pi) homeostasis in plant. This study was to genome-wide identification and analysis of *Solanum lycopersicum* SPX-domain-containing protein gene family. The *Solanum lycopersicum* genome contains 19 SPX-domain-containing protein genes. These SPX-domain-containing protein genes were located in seven of the 12 chromosomes. According to the different conserved domains, the proteins encoded by those genes could be divided into four SPX-domain-containing protein families, which included SPX Family, SPX-ERD1/XPR1/SYG1(SPX-EXS) Family, SPX-Major Facilitator Superfamily (SPX-MFS) Family and SPX-Really Interesting New Gene (SPX-RING) Family. Phylogenetic analysis of SPX-domain-containing protein genes in *Arabidopsis thaliana*, *Solanum tuberosum*, *Capsicum annuum* and *Solanum lycopersicum* classified these genes into eight clades. Expression profiles derived from transcriptome (RNA-seq) data analysis showed 19 SPX-domain-containing protein genes displayed various expression patterns. SPX-domain-containing protein may play different roles in phosphate nutrition of *Solanum lycopersicum* different tissues and development stages. And, this study can provide the selection of candidate genes for functional research and genome editing in *Solanum lycopersicum* phosphate ions (Pi) nutrition.

## Introduction

Proteins containing the Syg1/Pho81/XPR1 (SPX) domain play an important role in maintaining phosphate ions (Pi) homeostasis at the cell level (Duan et al., 2008; Hamburger et al., 2002; Wang et al., 2009b). In yeast, the SPX domain of Pho87 and Pho90 modulated Pi uptake activity and affected the regulation of the Pi signaling pathway (Hürlimann et al., 2009). In plants, SPX-domain-containing proteins are crucial to Pi homeostasis and signaling transduction (Zhang et al., 2016). The four families of SPX-domain-containing proteins have been found in plant, including SPX Family, SPX-ERD1/XPR1/SYG1 (EXS) Family, SPX-Major Facilitator Superfamily (MFS) Family and SPX-Really Interesting New Gene (RING) Family (Hamburger et al., 2002; Kant, Peng & Rothstein, 2011; Lin et al., 2010; Secco, Baumann & Poirier, 2010; Stefanovic et al., 2007; Wang et al., 2012). The Arabidopsis genome encodes 20 SPX-domain-containing proteins, grouped into SPX Family, SPX-EXS Family, SPX-MFS Family and SPX-RING Family (Duan et al., 2008).

There are four members of the SPX family in Arabidopsis, named AtSPX1, AtSPX2, AtSPX3, and AtSPX4. AtSPX1 and AtSPX3 are positive regulators in plant adaptation to phosphate starvation, and AtSPX1 and AtSPX3 act as negative regulators of some phosphate starvation induced (PSI) genes (Duan et al., 2008; Zhang et al., 2016). In rice, six members of the SPX family have been found, named OsSPX1, OsSPX2, OsSPX3, OsSPX4, OsSPX5 and OsSPX6 (Wang et al., 2009a; Liu, Wang & Ren, 2010; Wang et al., 2009b). In soybean, 10 members of the SPX family have been found, named GmSPX1, GmSPX2, GmSPX3, GmSPX4, GmSPX5, GmSPX6, GmSPX7, GmSPX8, GmSPX9 and GmSPX10 (Zhang et al., 2016; Yao, Tian & Liao, 2014).

In vascular (*Arabidopsis thaliana*) and non-vascular (*Physcomitrella patens*) plants, SPX-EXS family plays important roles in the acquisition, translocation and allocation of phosphate (Wang et al., 2004; Wang, Secco & Poirier, 2008; Zhao, You & Lei, 2019). The genome of Arabidopsis contains 11 members of SPX-EXS family, named AtPHO1 family, including AtPHO1, AtPHO1;H1, AtPHO1;H2, AtPHO1;H3, AtPHO1;H4, AtPHO1;H5, AtPHO1;H6, AtPHO1;H7, AtPHO1;H8, AtPHO1;H9 and AtPHO1;H10 (Wang et al., 2004). PHO1 family plays a broad role in inorganic phosphate homeostasis in Arabidopsis, such as transfer phosphate to the vascular cylinder of tissues of roots, leaves, stems, or flowers and acquisition phosphate into cells of pollen or root epidermal/cortical (Wang et al., 2004). Seven members of the SPX-EXS family have been identified in nonvascular plants of the moss *Physcomitrella patens*, named PpPHO1 family, including PpPHO1;1, PpPHO1;2, PpPHO1;3, PpPHO1;4, PpPHO1;5, PpPHO1;6, PpPHO1;7 (Wang, Secco & Poirier, 2008). PpPHO1 family has different, yet overlapping, functions in Pi transport and/or Pi homeostasis in protonemata, leaves, rhizoids, and auxiliary hairs of the moss (Wang, Secco & Poirier, 2008). The tomato (*Solanum lycopersicum*) genome contains six members of SPX-EXS family, named SlPHO1;1, SlPHO1;2, SlPHO1;3, SlPHO1;4, SlPHO1;5, SlPHO1;6 (Zhao, You & Lei, 2019). A CRISPR/Cas9 deletion into the phosphate transporter SlPHO1;1 indicates that SlPHO1;1 plays an important role in phosphate nutrition in the tomato seedling stage (Zhao, You & Lei, 2019).

SPX-MFS family harbors a SPX domain and an MFS domain (Lin et al., 2010). SPX-MFS family is a new group of vacuolar Pi transporters in plants (Liu et al., 2016a, 2016b; Wang et al., 2015). *Arabidopsis thaliana* has three members of SPX-MFS family, designated as PHT5 family, and PHT5 family consists of AtPHT5;1, AtPHT5;2 and AtPHT5;3 (Liu et al., 2016a). Arabidopsis PHT5 proteins contribute to the Pi import into vacuoles mediating phosphate storage (Liu et al., 2016a, 2016b). In rice, there are four members for SPX-MFS family, namely OsSPX-MFS1, OsSPX-MFS2, OsSPX-MFS3 and OsSPX-MFS5 (Lin et al., 2010; Secco et al., 2012). The rice OsSPX-MFS3 was responsible for the vacuolar Pi export and OsSPX-MFS1 contribute to the Pi import into vacuoles (Liu et al., 2016a; Wang et al., 2015).

SPX-RING Family members contain both the N-terminal SPX and C-terminal RING domains (Lin, Huang & Chiou, 2013). In Arabidopsis, the only member of the SPX-RING family called Nitrogen Limitation Adaptation (NLA) protein, which was initially shown to function in nitrogen limitation responses (Peng et al., 2007). Another more recent study showed that NLA was related to Pi responses and involve in phosphate homeostasis (Kant, Peng & Rothstein, 2011). Two SPX-RING Family members from rice (*Oryza sativa* L.) were identified and designated *OsNLA1* and *OsNLA2* (Yang et al., 2017). OsNLA1 has pivotal roles in mantaining Pi homeostasis in rice (Yue et al., 2017; Zhong et al., 2017).

The domesticated tomato (*Solanum lycopersicum*) is a major vegetable crop worldwide and a model plant for biological and genetic research of fruit development, domestication and stress responses (Tomato Genome Consortium, 2012; Klee & Giovannoni, 2011; Tieman et al., 2017; Bai et al., 2018). For plant, phosphorus is an essential macronutrient, and plays a key role in growth and development processes (Chapin, 1980; Raghothama, 1999; Vance, 2001). In plant, as an element of nucleic acids, phospholipids and primary metabolites, phosphorus is critically important for plant cell structure and function, and affects almost all physiological reactions such as carbohydrate metabolism, glycolysis, photosynthesis, respiration, nucleic acid synthesis and redox reactions (Zhang, Liao & Lucas, 2014; Marschner, 1995). In addition, as a regulator, phosphorus participates in enzymatic reactions and signal transduction processes (Linn et al., 2017). Tomato production needs a lot of phosphorus, and SPX-domain-containing protein gene family plays important roles in the acquisition, translocation, allocation of phosphate and Pi signaling pathway, so it is very necessary for genome-wide identification and characterization of SPX-domain-containing protein gene family in *Solanum lycopersicum*. In this study, we identified 19 SPX-domain-containing protein genes representing all four families (SPX Family, SPX-EXS Family, SPX-MFS Family and SPX-RING Family).

In addition, we conduct a systematic analysis of the 19 SPX-domain-containing protein genes, including the prediction of gene structures, phylogenetic relationships, conserved motifs, chromosomal distributions and expression patterns of these genes. All these results contribute to a deeper understanding of their potential critical roles in maintaining Pi homeostasis. Thus, our results provided the basis for further research into the Pi homeostasis and signaling transduction processes functions of the SPX-domain-containing proteins in tomato, and help further genetic modification of tomato to improve Pi use efficiency.

## Methods

### Identification of SPX-domain-containing proteins in *Solanum lycopersicum*

To identify SPX-domain-containing proteins in the *Solanum lycopersicum* genome, “SPX” was employed to search UniProt (https://www.uniprot.org) databases. And then, BLASTp searches were performed employing the SPX corresponding protein sequences from Arabidopsis against the SGN (https://solgenomics.net/) databases. Finally, all the retrieved non redundant hypothetical protein sequences were submitted to the Pfam database (http://pfam.xfam.org/) (EI-Gebali et al., 2019) and the SMART website (http://smart.embl-heidelberg.de/) (Letunic & Bork, 2018) to identify SPX domain. The same procedure was used to search for SPX-domain-containing proteins in *Arabidopsis thaliana*, *Solanum tuberosum*, *Capsicum annuum*.

### SPX-domain-containing protein properties, chromosomal distribution, and gene structure analysis

Each SPX-domain-containing protein sequence were submitted to the ExPaSy (http://expasy.org/) (Gasteiger et al., 2005) to compute the molecular weight (MW) and isoelectric point (pI). To analyze chromosomal distribution of SPX-domain-containing protein family genes, each SPX-domain-containing protein family gene was mapped to the chromosomes by the SGN (https://solgenomics.net/) databases. The structures of the SPX-domain-containing protein genes were predicted using the online tool GSDS (http://gsds.gao-lab.org/) (Hu et al., 2015).

### Phylogenetic tree construction and the domains analysis of the SPX-domain-containing protein genes

Phylogenetic tree was constructed with the MEGA 7.0 software (Kumar, Stecher & Tamura, 2016) using the neighbour-joining (NJ) method with 1,000 bootstrap replicates. And the high-quality figure for phylogenetic tree of the SPX-domain-containing protein genes was created using the online tool ITOL (https://itol.embl.de).

The protein domains in the SPX-domain-containing protein family were identified using the online program Pfam (http://pfam.sanger.ac.uk/) (EI-Gebali et al., 2019) and the domain figures were generate using MyDomains-Image Creator (https://prosite.expasy.org/) (Hulo et al., 2008).

### Expression analysis of *Solanum lycopersicum* SPX-domain-containing protein genes in different tissues

The transcriptome data (RNA-seq) of gene expression in different tissues including bud, flower, leaf, root, 1 cm fruit, 2 cm fruit, 3 cm fruit, mature green, breaker, and breaker after 10 days were downloaded from the Tomato Functional Genomics Database (http://ted.bti.cornell.edu/). The expression profiles of the *Solanum lycopersicum* SPX-domain-containing protein genes were estimated by RPKM values (reads per kilobase per million mapped reads) and a heat map was constructed to show the different expression profiles by the TBtools software (version No. 0.6739) (Chen et al., 2020).

## Results

### Identification of SPX-domain-containing Proteins in *Solanum lycopersicum*

Based on the UniProt (https://www.uniprot.org) databases, the SGN (https://solgenomics.net/) databases, the Pfam database (http://pfam.xfam.org/) and the SMART website (http://smart.embl-heidelberg.de/), a total of 19 SPX-domain-containing proteins were identified (Table 1). In the 19 SPX-domain-containing proteins, the length of the protein-coding regions ranged from 573 bp (Solyc00g149970) to 3,735 bp (Solyc08g080200), and the proteins ranged from 190 to 1,244 amino acids (aa) in length. The predicted molecular weights (MWs) of the 19 SPX-domain-containing proteins varied from 21.16 kDa to 138.04 kDa, and the isoelectric points (pI) ranged from 4.68 to 9.56.

**Table 1:** Description of *Solanum lycopersicum* SPX-domain-containing proteins family genes.

Gene ID	Location	Strand	ORF (aa)	CDS (bp)	MW (KDa)	pI
Solyc00g149970	chr0	unknown	190	573	21.16	9.56
Solyc01g090890	chr1:84470111–84472549	Plus	262	789	29.66	8.79
Solyc01g091870	chr1: 85284503–85301327	Plus	1,001	3,006	112.15	5.75
Solyc02g067160	chr2: 37919619–37929781	Minus	307	924	34.44	4.9
Solyc02g088210	chr2: 51007852–51014337	Plus	296	891	33.58	4.68
Solyc02g088230	chr2: 51007870–51031092	Minus	792	2,379	92.27	8.67
Solyc02g088220	chr2: 51036940–51040775	Minus	777	2,334	89.98	9.35
Solyc02g088250	chr2: 50401478–50403738	Minus	414	1,245	47.54	9.14
Solyc05g010060	chr5: 4268586–4273832	Plus	787	2,364	92.04	9.17
Solyc05g013180	chr5: 6257498–6263752	Minus	797	2,394	93.48	9.34
Solyc08g007800	chr8: 2311077–2321989	Minus	694	2,085	77.8	6.25
Solyc08g060920	chr8: 45846503–45848017	Plus	266	801	30.76	5.96
Solyc08g068240	chr8: 57408077–57412791	Plus	814	2,445	94.39	9.16
Solyc08g080200	chr8: 63633650–63649570	Minus	1,244	3,735	138.04	6.4
Solyc09g075040	chr9: 67259148–67263721	Minus	333	1,002	37.96	8.95
Solyc09g090360	chr9: 70337479–70350032	Minus	788	2,367	90.82	9.21
Solyc11g045230	chr11: 31612612–31618788	Minus	335	1,008	38.3	8.63
Solyc12g009480	chr12: 2734650–2737850	Minus	292	879	33.56	5.65
Solyc12g056440	chr12: 63312770–63324987	Plus	697	2,094	78	5.53

**Figure 1:**
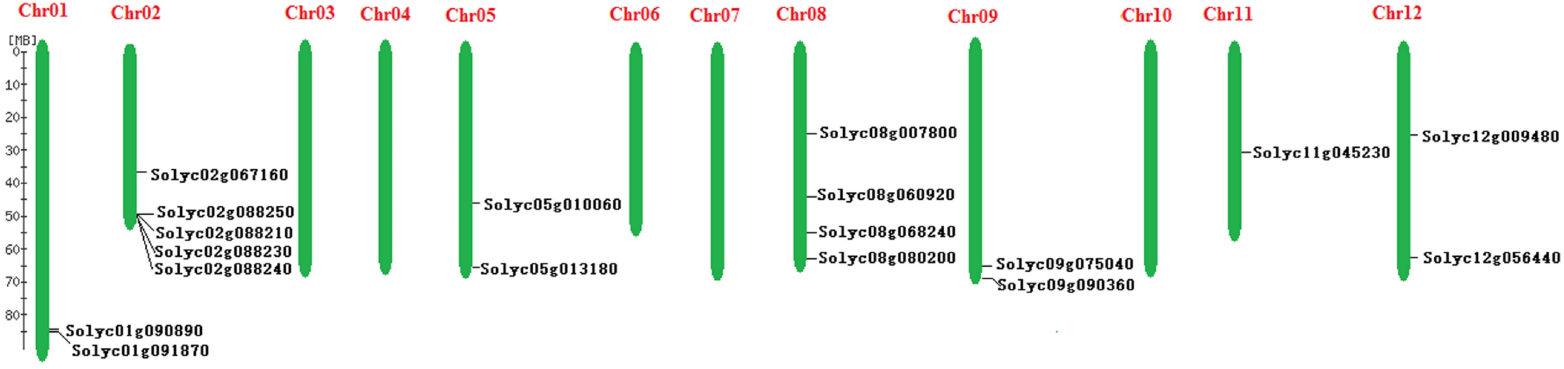
Chromosomal locations of the SPX-domain-containing proteins family genes in the *Solanum lycopersicum*.

The chromosomal distribution of the *Solanum lycopersicum* SPX-domain-containing proteins genes was indicated in Fig. 1. Of the 19 SPX-domain-containing proteins genes in *Solanum lycopersicum*, 18 were successfully mapped to seven of the 12 *Solanum lycopersicum* chromosomes, while one SPX-domain-containing proteins gene, Solyc00g149970, was located on chr0. Two each SPX-domain-containing protein genes were located on chr1, chr5, chr9, chr12. Five SPX-domain-containing proteins genes were located on chr2, four on chr8, and only one SPX-domain-containing proteins gene assigned to chr11 (Fig. 1).

### Phylogenetic analysis of the SPX-domain-containing proteins genes in the *Solanum lycopersicum* and other species

**Figure 2:**
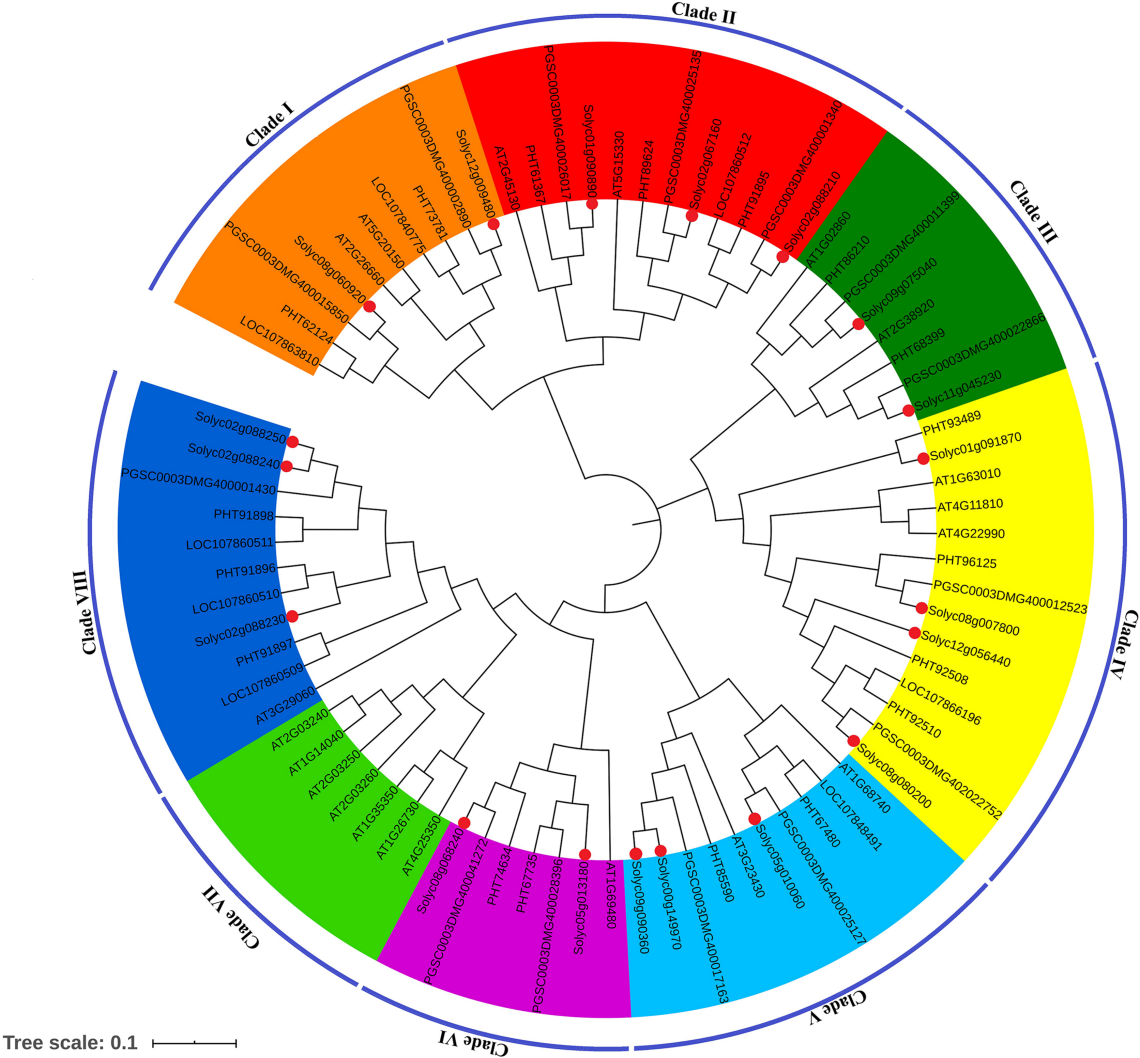
Phylogenetic analysis of the SPX-domain-containing protein genes in *Arabidopsis thaliana* (At), *Solanum tuberosum* (PGSC), *Capsicum annuum* (PHT and LOC) and *Solanum lycopersicum* (Solyc). Phylogenetic tree was constructed with the NJ algorithm and 1,000 bootstrap replicates. Differently colors indicate different groups (or subgroups) of SPX-domain-containing protein genes. Red dots indicate *Solanum lycopersicum* SPX-domain-containing protein genes.

To evaluate evolutionary relationships of SPX-domain-containing protein genes in *Solanum lycopersicum*, we analyzed the sequence features in four different species, including *Arabidopsis thaliana*, *Solanum tuberosum*, *Capsicum annuum* and *Solanum lycopersicum*, and a total of 79 SPX-domain-containing protein genes were used to construct a phylogenetic tree with the neighbor-joining (NJ) method using MEGA (version 7) (Fig. 2). As shown in this phylogenetic tree, all SPX-domain-containing protein genes were divided into eight subfamilies: Clade I, Clade II, Clade III, Clade IV, Clade V, Clade VI, Clade VI and Clade VIII. All the eight subfamilies can be grouped into four bigger subfamilies: SPX, SPX-EXS, SPX-MFS and SPX-RING. Clade I and Clade II were comprised of the SPX family members, Clade III was comprised of the SPX-RING family members, Clade IV was mainly comprised of the SPX-MFS family members, and Clade V, Clade VI, Clade VI and Clade VIII were mainly comprised of SPX-EXS family members.

### Structures analysis of the SPX-domain-containing genes and proteins in the *Solanum lycopersicum*

**Figure 3:**
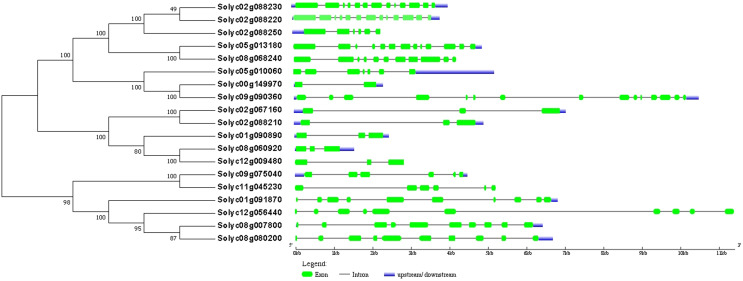
Phylogenetic relationships and structural analysis of SPX-domain-containing genes in *Solanum lycopersicum*. Exons and introns are represented by green boxes and black lines, respectively. Lengths of exons and introns of each SPX-domain-containing gene are exhibited proportionally.

The phylogenetic relationships and structural analysis of the 19 SPX-domain-containing genes showed that the members within each subfamily (SPX-MFS and SPX-RING) had the same exon–intron structures, but the members within each subfamily (SPX and SPX-EXS) showed a certain degree of complexity (Fig. 3). The SPX-MFS subfamily (Solyc01g091870, Solyc08g007800, Solyc08g080200 and Solyc12g056440) had 10 exons. Members of the SPX-RING subfamily possessed six exons, while SPX subfamily (Solyc00g149970, Solyc02g088250, Solyc08g060920, Solyc01g090890, Solyc12g009480, Solyc02g067160 and Solyc02g088210)—except for Solyc00g149970 & Solyc02g088250, which had two & six—possessed three. In the SPX-EXS subfamily (Solyc02g088230, Solyc02g088220, Solyc05g010060, Solyc05g013180, Solyc08g068240 and Solyc09g090360), three members (Solyc02g088220, Solyc02g088230 and Solyc05g013180) had 13 exons, one (Solyc08g068240) had 11 exons and one member (Solyc09g090360) had 15.

**Figure 4:**
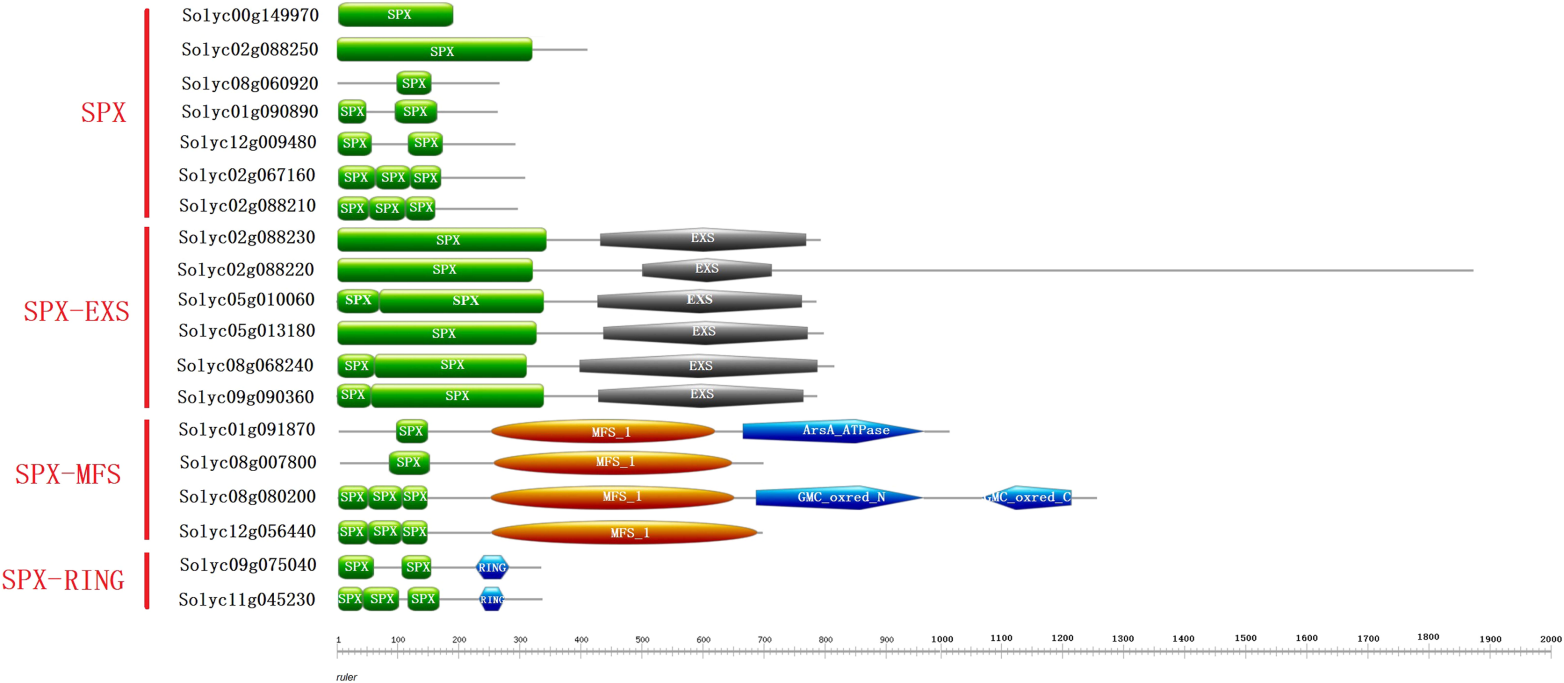
Domains of the SPX-domain-containing proteins in the *Solanum lycopersicum*. Conserved domains of the SPX-domain-containing proteins were marked with different colors and shapes. The online program Pfam was used to identify the conserved domains in the different proteins.

All the 19 SPX-domain-containing proteins contain SPX domain in the C-terminal portion (Fig. 4). The four families of SPX-domain-containing proteins (SPX, SPX-EXS, SPX-MFS and SPX-RING) have been found in *Solanum lycopersicum*. The SPX family possessed eight members, SPX-EXS family contained five members, SPX-MFS family had four members, and SPX-RING family possessed two members.

### Expression analysis of *Solanum lycopersicum* SPX-domain-containing protein genes in different tissues

**Figure 5:**
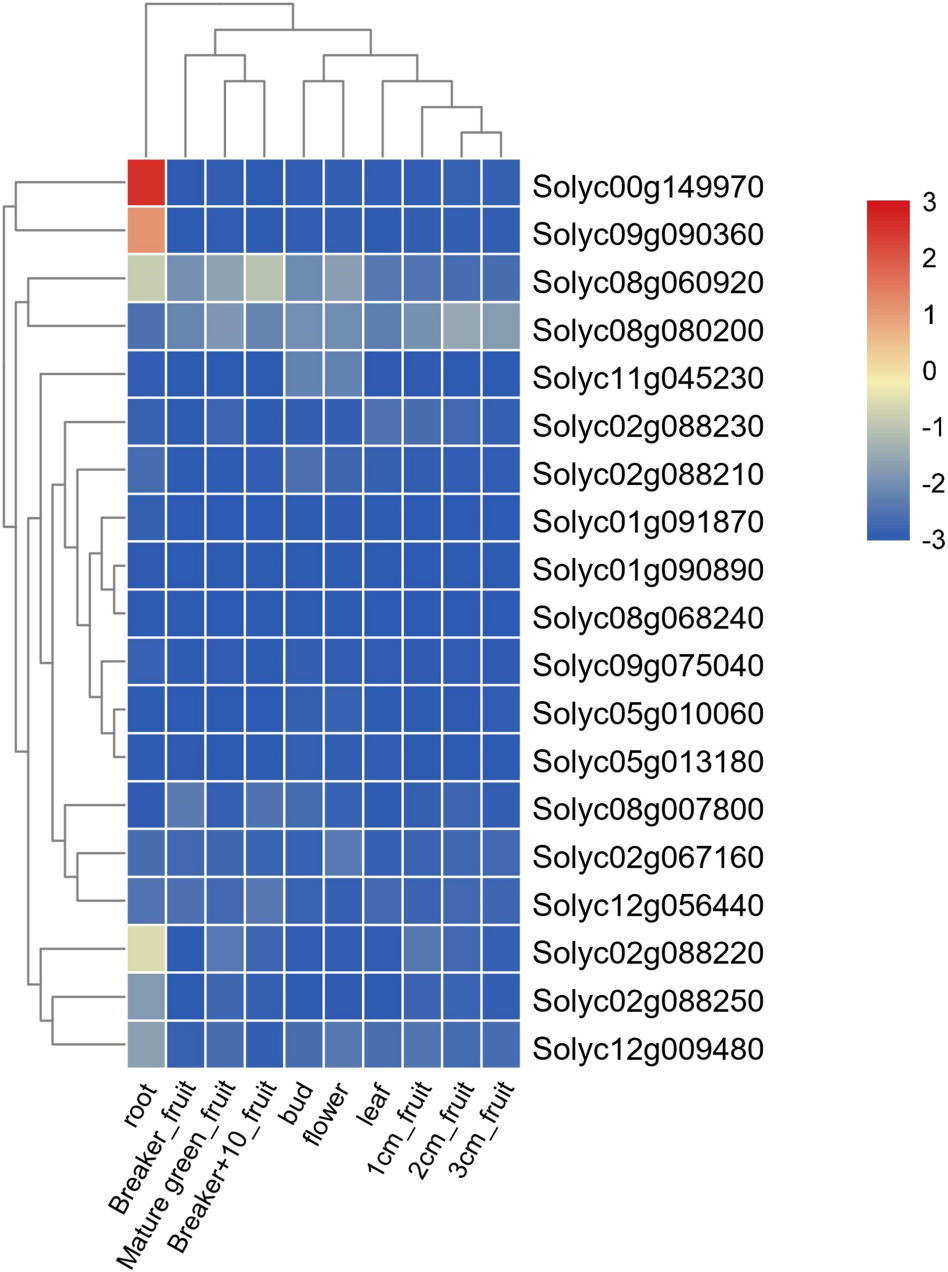
Expression profile of 19 *Solanum lycopersicum* SPX-domain-containing protein genes in different tissues and fruit developmental stages based on transcriptome (RNA-seq) data. RPKM-normalized values from RNA-seq data for different tissues of the *Solanum lycopersicum* were used to construct the heat map. The SPX-domain-containing protein genes are divided into four groups (SPX, SPX-EXS, SPX-MFS and SPX-RING). The scale representing the relative signal values is at the right upper corner of the heatmap.

To analyze expression profiles of the 19 *Solanum lycopersicum* SPX-domain-containing protein genes in different tissues and fruit developmental stages, we constructed a heat map using a published tomato RNA-seq dataset from the Tomato Functional Genomics Database (http://ted.bti.cornell.edu/) (Fig. 5, Tables S1–S5). The SPX family contained eight genes and about half of them were strongly expressed in root tissue, one member (Solyc08g060920) was expressed in bud, flower, root, mature green_fruit, breaker_fruit and breaker+10_fruit, but was preferentially expressed in root tissues and the fruit developmental stage of breaker after 10 days (Fig. 5, Tables S4, S5). In SPX-EXS family, two members were dominantly expressed in root tissues, implying that they may be important for Pi homeostasis and transfer in roots tissues (Fig. 5, Tables S4, S5). The SPX-MFS family included four genes, one member (Solyc08g080200) was expressed in bud, flower and different fruit developmental stages, and they had different expression levels in different fruit developmental stages (Fig. 5, Tables S4, S5). Another member of the SPX-MFS family was preferentially expressed in breaker_fruit and breaker+10_fruit stages (Fig. 5, Tables S4, S5). One member of the SPX-RING family showed high expressions in bud and flower, implying that it may play key roles for Pi homeostasis and transfer in bud and flower tissues (Fig. 5, Tables S4, S5).

## Discussion

SPX-domain-containing proteins are vital in plant maintaining Pi homeostasis at the cell level (Duan et al., 2008; Hamburger et al., 2002; Wang et al., 2009b). In plants, SPX-domain-containing proteins can be divided into four families: SPX family, SPX-EXS family, SPX-MFS family and SPX-RING family (Secco et al., 2012). The *Solanum lycopersicum* genome encodes 19 SPX-domain-containing proteins, covered all of the four families including SPX Family, SPX-EXS Family, SPX-MFS Family and SPX-RING Family.

SPX Family could perform the regulation functions of Pi uptake and mobilization in plant (Duan et al., 2008; Osorio et al., 2019; Wang et al., 2009a, 2009b). The Arabidopsis SPX family consists of four members named AtSPX1-AtSPX4 (Duan et al., 2008), and the rice genome contains six members of the SPX family designated as OsSPX1-OsSPX6 (Wang et al., 2009a). In *Solanum lycopersicum* genome, we identified seven SPX family members (Solyc00g149970, Solyc02g088250, Solyc08g060920, Solyc01g090890, Solyc12g009480, Solyc02g067160 and Solyc02g088210). The SPX family genes have the similar gene structure with three exons and two introns between rice and Arabidopsis, except for OsSPX5, which possessed two exons and one intron (Duan et al., 2008; Wang et al., 2009a). In the SPX family members of *Solanum lycopersicum*, five members (Solyc08g060920, Solyc01g090890, Solyc12g009480, Solyc02g067160 and Solyc02g088210) have the similar gene structure with Arabidopsis, and the rest two (Solyc00g149970 and Solyc02g088250) were different from Arabidopsis. Further analysis indicates that the gene structure of Solyc00g149970 was similar to OsSPX5, while Solyc02g088250 was different from Arabidopsis and rice, which possessed six exons and five introns. The SPX family protein structures are more divergent in *Solanum lycopersicum*, which have one, two or even three SPX domains. Various expression patterns for SPX family genes were observed in rice and Arabidopsis (Duan et al., 2008; Wang et al., 2009a). The SPX family genes of *Solanum lycopersicum* displayed also various expression patterns, but about half of them were strongly expressed in root tissue.

Some members of the SPX-EXS Family could perform Pi loading in the xylem or play an important role in the root-to-shoot Pi-deficiency signaling network (Secco et al., 2012). In Arabidopsis, the SPX-EXS family consists of 11 members, namely AtPHO1–AtPHO1;H10 (Wang et al., 2004). Existed studies have shown that AtPHO1 and AtPHO1;H1 were involved in Pi loading to the xylem (Stefanovic et al., 2007). In rice, the SPX-EXS family consists of three members, namely OsPHO1;1–OsPHO1;3, and OsPHO1;2 plays a key role in the transfer of Pi from roots to shoots (Secco, Baumann & Poirier, 2010). *Physcomitrella patens* is a kind of non-vascular plant, of which genome contains seven members of SPX-EXS family, named PpPHO1;1–PpPHO1;7, and some members play distinct functions in Pi transport and/or Pi homeostasis (Wang, Secco & Poirier, 2008). In *Solanum lycopersicum* genome, there are six members of SPX-EXS family, named SlPHO1;1–SlPHO1;6, and a CRISPR/Cas9 deletion into SlPHO1;1 indicates that SlPHO1;1 plays a key role in phosphate nutrition in *Solanum lycopersicum* seedling stage (Zhao, You & Lei, 2019). SPX-EXS family showed various expression patterns in Arabidopsis (Wang et al., 2004). In Rice, the three members (OsPHO1;1–OsPHO1;3) of SPX-EXS family also showed various expression patterns, but two members (OsPHO1;1 and OsPHO1;2) were strongly expressed in roots (Secco, Baumann & Poirier, 2010). In non-vascular plant, SPX-EXS family has the distinct expression patterns in various tissues (Wang, Secco & Poirier, 2008). The transcriptome (RNA-seq) data revealed that SPX-EXS family of *Solanum lycopersicum* has the similar expression patterns with Arabidopsis, Rice and *Physcomitrella patens*, and two members (Solyc02g088220 and Solyc09g090360) were dominantly expressed in root tissues.

SPX-MFS family performs Pi remobilization in the leaves (Secco et al., 2012). The Arabidopsis SPX-MFS family consists of three members, namely AtPHT5;1–AtPHT5;3, which function as vacuolar Pi (vac-Pi) importers (Liu et al., 2016a). Rice has four members for SPX-MFS family, namely OsSPX-MFS1–OsSPX-MFS4, and OsSPX-MFS3 as a vacuolar phosphate efflux transporter mediates Pi efflux from the vacuole into cytosol (Wang et al., 2015). In *Solanum lycopersicum*, we identified four members for SPX-MFS family, but their functions were not clear. *AtPHT5;1* and *AtPHT5;3* showed a similar expression pattern which were expressed in most tissues, while *AtPHT5;2* was expressed in guard cells, vascular tissue and pollen (Liu et al., 2016a).

The three *OsSPX-MFS* genes were expressed in leaf, shoot, root, flower and panicle, and *OsSPX-MFS3* was expressed significantly higher than *OsSPX-MFS1* and *OsSPX-MFS2* (Wang et al., 2015). The four SPX-EXS family genes of *Solanum lycopersicum* were also expressed in various tissues, including bud, flower, root, mature green_fruit, breaker_fruit and breaker+10_fruit, and one member of them (*Solyc08g080200*) had some similarities with OsSPX-MFS3 such as expressing significantly higher than others in various tissues.

In Arabidopsis, the only member of the SPX-RING family was identified, named NLA, which was not only related to nitrogen limitation responses but also involved in phosphate homeostasis (Peng et al., 2007; Kant, Peng & Rothstein, 2011). Two members of SPX-RING family were identified in rice, namely OsNLA1 and OsNLA2, and OsNLA1 has pivotal roles in mantaining Pi homeostasis (Yang et al., 2017; Yue et al., 2017; Zhong et al., 2017). The *Solanum lycopersicum* SPX-RING family consists of two members, and the functions about Pi homeostasis need to be explored in depth. *AtNLA* was expressed higher in root and stem than in seedling, flower, rosette and cauline leaves, and lower than in siliques (Peng et al., 2007). *OsNLA1* was expressed in leaf, root, stem, sheath, open inflorescence and closed spikelets, and the greatest transcript levels were detected in the sheath and leaf tissues (Zhong et al., 2017). The two members (Solyc09g075040 and Solyc11g045230) of *Solanum lycopersicum* SPX-RING family were expressed in bud, flower, root, mature green_fruit, breaker_fruit and breaker+10_fruit, and *Solyc11g045230* were expressed higher in bud and flower.

## Conclusions

A total of 19 SPX-domain-containing protein genes were identified in *Solanum lycopersicum* genome. In *Solanum lycopersicum*, all the four families of SPX-domain-containing proteins (SPX, SPX-EXS, SPX-MFS and SPX-RING) had been found. The results of expression analysis showed that the 19 SPX-domain-containing protein genes displayed various expression patterns. The above results implied that they may play different roles in phosphate nutrition of *Solanum lycopersicum* different tissues and development stages. These results provide references for the further study of *Solanum lycopersicum* SPX-domain-containing proteins family genes. Moreover, this study provides the selection of candidate genes for functional research and genome editing in *Solanum lycopersicum* phosphate nutrition.

### Data Availability statement

The data that support this study are available in the Tomato Functional Genomics Database (http://ted.bti.cornell.edu/).

## Supplemental Information

10.7717/peerj.12689/supp-1 Supplemental Information 1 Supplementary Tables.

## Abbreviations

SPX: SYG1, PHO81, and XPR1
SPX-EXS: SPX-ERD1/XPR1/SYG1
SPX-MFS: SPX-Major Facilitator Superfamily
SPX-RING: SPX-Really Interesting New Gene
Pi: Phosphate ions
PSI: Phosphate starvation induced
aa: Amino acids; in length
MWs: Molecular weights
Pi: Isoelectric points
NJ: Neighbor-joining

## Additional Information and Declarations

### Data Availability

The following information was supplied regarding data availability: The raw measurements are available in the Supplementary File.
